# Dr. Cooke on Epilepsy

**Published:** 1823-06-01

**Authors:** 


*History and Method of Cure of the Various Species of Epilepsy: being the Second Part of the Second Volume of a Treatise on Nervous Diseases.* By John Cooke, M.D. F.R.S. F.A.S. Fellow of the Royal College of Physicians, and late Physician to the London Hospital.

Dr. Cooke pursues his erudite and industrious researches, and with the subject of epilepsy concludes his second volume of nervous diseases. It were greatly to be wished that a few of our Nestors in medecine would each select a class of diseases for their researches and meditations, in the manner of Dr. Cooke, in order that the general mass of professional society might become acquainted with the opinions and practices of ancient and modern writers, at a much easier rate than they now can. In fact, without some such auxiliary as this, ninety-nine in the hundred of our brethren must live and die in complete ignorance of the stores of medical literature now buried in the tomes of antiquity. Such an undertaking therefore as that of Dr. Cooke, deserves two things—encouragement by the public, and imitation by some of his learned brethren. But, without further preface, we must proceed to the volume before us, which appears to be constructed with equal skill and judgement as the preceding parts of the Treatise.

The work is divided into five chapters, embracing the definition and history, dissections, distinctions and causes, diagnosis and prognosis, and lastly, the treatment of epilepsy. These we shall notice seriatim, though very concisely.*

1. *Definition and History.* The strange and formidable phenomena presented in epilepsy early attracted the attention of mankind, and called forth extraordinary appellations, as morbus sacer, comitialis, herculeus, caducus, &c. some of which are fanciful, some characteristic, and others enigmatical. The following is Dr. Cooke's definition.

"It is a disease consisting of paroxysms of convulsion, returning at uncertain periods, accompanied by an abolition of sense and voluntary motion, and ending in somnolency or complete sleep." 5.

We acknowledge the difficulty of definitions of disease, and therefore seldom criticise them. It appears to us, however, that the above definition is somewhat redundant, if indeed it be strictly correct. If we see the *voluntary muscles* in strong action, (whether with or without the will) can we strictly say that there is an "*abolition* of voluntary motion." ? That there is *involuntary* motion admits of no doubt, and this being expressed by the term "convulsions," we think the "abolition," &c. superfluous.† But this is of no consequence.

---

* The matter in this volume, is still more densely concentrated than in any of the preceding—a circumstance that renders it difficult, or indeed impossible to give a closely connected chain of analysis—but which greatly enhances the value of the work to the public.

† We are strongly disposed to coincide in opinion with the physician alluded to in the following note at page 10 of the volume before us.

"But a physician, to whose opinions I pay great respect, informs me, that he doubts very much whether the will be always inactive in epilepsy, and whether the muscles are not to a certain degree obedient to it. The resistance to the exertions, he says, made by the bystanders to confine the limbs, appears to me, in some cases, to be voluntary, and to argue some perception of that resistance, and some desire to overcome it. The state of mind, in these cases, appears to me like that which takes place in dreaming and in delirium." 10.

The epileptic attack is more frequently without than with premonitory symptoms. When the latter take place, they are various and uncertain, as languor, pain or giddiness of the head, disturbed sleep, dimness of sight, tinnitus aurium, &c. Areteus enumerates distention of the veins of the neck, nausea and vomiting, flashes of light before the eyes, sense of disagreeable odours, &c. But the most remarkable precursor is the aura epileptica, a sensation difficult to be described, being likened by some to a stream of cool and gentle air blowing on the part affected, or ascending to the head from one of the extremities, when the paroxysm immediately takes place. Not the least light has yet been thrown on this mysterious sensation by any physiologist, though we can hardly attribute it to any other medium than the nervous system. In many instances it has distinctly arisen from a local irritation on the extremity of a nerve. We need not dwell on the symptoms of the fit. They have been described by poets and physicians of all ages. Lucretius has given a very spirited sketch of the epileptic paroxysm from which, however, we shall only extract two or three lines as an example.

> " ——————— ut fulminis ictu,
> Concidit, et spumas agit, ingemit, et tremit artus,
> Desipit, extentat nervos, torquetur, anhelat,
> Inconstanter et in jactando membra fatigat."

The terminations of epilepsy are various—as apoplexy, paralysis, particularly of the nerves of hearing and vision—but its most common termination is in idiotism or fatuity.

2. *Morbid Anatomy.* The morbid appearances presented in the persons of epileptics are chiefly confined to the brain, and many of them are very similar to those found after apoplexy or paralysis. These are generally effusions and tumours of various kinds—abscesses—exostoses—depressed portions of bone—sharp-pointed spiculæ, &c. which mechanically press upon and injure the brain or its investing membranes. The blood-vessels of the encephalon are very generally found greatly distended—and even blood itself has been often found extravasated—but not so frequently as serous fluid. A jellatinous substance—deep seated collections of pus—and a great variety of other morbid appearances have been discovered in the brains of epileptics, which it would be impossible even to enumerate.

It is strange, as Dr. Cooke remarks, that in the accounts of morbid appearances found after epilepsy, little or no notice is taken of the state of the cerebellum. Yet a celebrated modern physiologist confidently asserts that, on examinations made by him, the *cerebellum* has *always* been found very greatly diseased. The physiologist in question is Wenzel, of Mayence, whose opportunities for observation have been very extensive, and the result of whose enquiries has been published by his brother, under the title "*Observations sur le Cervelet, et sur les Diverses Parties du Cerveau, dans les Epileptiques.*" Wenzel, impressed with a conviction that no individual could much advance the pathology and treatment of epilepsy, instituted a society for the purpose of co-operation in this work, the members of which entered into his views, and prosecuted the investigation with zeal. We shall give a long extract from the work before us, as Wenzel's observations are nearly unknown in this country.

"M. Wenzel had an opportunity of examining the heads of twenty persons, who had died, after epilepsy of the worst kind, and he has minutely described many curious alterations of structure, singular effusions, and other marks of great and uncommon disease, or at least of disease not hitherto noticed, of parts within the cranium, which are particularly interesting.

"The brain, properly so called, has been generally considered as the chief seat of disease in epilepsy; M. Wenzel, however, in a very great proportion of the heads he examined, found that organ in a healthy state, but the cerebellum uniformly and in a very extraordinary degree and manner diseased. In fifteen of the twenty cases above mentioned, the brain was uninjured, but of the other five, in two the meninges were slightly diseased, in four, an effusion of a thick lymph was observed on the surface of the brain, and in three, a considerable quantity of water in the ventricles, in consequence of which the corpora striata and the thalami nervorum opticorum were much injured, and in one instance a softening and an enlargement of the brain was seen; but in fifteen of the twenty the brain was found to be in a sound state.

"The parts which M. Wenzel in these cases discovered to be principally affected, were the pineal gland, and the cerebellum; the former often, the latter always, injured in a greater or less degree. In ten instances the pineal gland was almost entirely of a grey colour; in one, it was white in its anterior part, and of a pale red in one half of its posterior part. In another case, on the superior surface of the pineal gland, a brownish yellow transparent vesicle was observed, from which a considerable quantity of a clear yellow fluid was thrown out; in a third, the pia mater surrounding the gland was thicker than ordinary, and partly of a red, partly of a yellow colour. The pineal gland was always found softer, and in all the instances, excepting two, smaller than natural; in those two, however, it was much enlarged.

"In three instances, the infundibulum was more firm than usual, and round about it, a thick lymph was effused, which in some places could be raised up like a membrane. The superior part of the infundibulum was in this case of a red colour, and bore marks of inflammation. In one instance, through its whole length, it was extremely red.

" The most extraordinary morbid appearances, however, discovered by M. Wenzel in these dissections, were those which he found in the cerebellum. He observed that organ to be morbidly affected with respect to its surface, its consistence, its colour, its size, and the state of its internal parts. In one case, the whole surface of the cerebellum appeared unequal and furrowed; in another, about the insertion of the infundibulum, there was a very large excavation, from a loss of the substance of almost the whole of its superior surface; and in a third, there was a great depression of the anterior edge of the cerebellum. In two instances, its superior surface was of a fibrous appearance, and much furrowed.

" The colour of the cerebellum was sometimes of a pale, but more commonly of a dusky red, of different shades, and approaching to blackness; sometimes it was of a whitish or yellow colour, and in two instances its posterior lobe was of a pale grey.

" In three cases, M. Wenzel found the cerebellum very soft, but in five others, it was harder and more compact than natural. Its size was often considerably increased; sometimes, under circumstances to be hereafter mentioned, it was prodigiously enlarged.

" The most remarkable and important alterations observed in these dissections were those found in the interior of the cerebellum, which had probably occasioned its increased size, and the diseased appearances on its surface, above mentioned.

" After having made one or two horizontal sections of the cerebellum, M. Wenzel, in ten instances out of the twenty, found between the lobes, at the point of their union, a yellow, friable, solid matter, which almost always had produced not only a separation of the lobes, but a loss of their substance. This matter could easily be raised in pieces. Sometimes, at the junction of the lobes, a half fluid viscous lymph was seen separating them; sometimes, upon the superior surface of the cerebellum, spots were produced, by a collection of a perfectly white or yellowish brown lymph, which had become solid. In those cases in which the cerebellum was observed to be much enlarged, a great quantity of lymph, more or less thick, was seen between the lobes. In one instance, on separating them, a large quantity of a thick colourless fluid was thrown out, which ran to the point of their union. M. Wenzel thinks, that if the subject of this dissection had lived longer, the fluid observed would have gradually acquired the appearance and colour of the matter seen in other cases, on examination after epilepsy. In one case, on cutting the cerebellum where the lobes touch, a round ball was observed, containing several small globular transparent bodies, much resembling the granulated substances often seen in the pineal gland. On the superior surface of the cerebellum, marks of inflammation were sometimes visible.

" These were the chief morbid appearances found by M. Wenzel, in the examinations of the brains of persons who had been affected with epilepsy." 38.

It is, as Dr. Cooke observes, truly surprising that Wenzel should have invariably found the cerebellum diseased in epilepsy, and that other pathologists should have scarcely noticed such a phenomenon. This mystery can only be settled by future investigations. The foregoing extract will induce our readers, when they have opportunities, to examine the cerebellum with great care, in their future dissections of epileptic patients.

It is not to be supposed, however, that the morbid appearances are confined entirely to the head, in epileptic cases. The heart, the lungs, the liver, and the intestines, have all been found in a morbid condition. M. Prost, in his "Medecine Eclairée par l'Observation et l'Ouverture des Corps," asserts that, in all the cases of epilepsy which he has examined, he found masses of worms in the intestines, generally accompanied with acrid substances in different parts of the alimentary canal. The spinal marrow has been found diseased in a considerable proportion of epileptics. Finally, some people have died of this disease where (it is said) no morbid appearances whatever presented themselves on dissection. This we can hardly believe; because we know how slovenly some dissections are performed. Not that we suppose it is absolutely necessary there should exist an organic affection of the brain or other part, in order that the phenomena of epilepsy may be developed. But we believe that few cases go on long, or prove fatal, without leaving traces of disorder in the vessels or nerves, or other structures of the body.

3. *Distinctions and Causes.* Dr. Cooke considers the disease under the two general heads, *idiopathic* and *symptomatic*—the first, a primary affection of the brain—the latter, having its origin in some other part of the system, the sensorium being affected secondarily.

In respect to etiology, Dr. Cooke believes, and with reason, that a certain constitution of brain and nervous system renders particular individuals predisposed to the attacks of epilepsy. This is the temperament "qui colligit et ponit iram temere, et mutatur in horas." A certain conformation of cranium seems to predispose to epilepsy. Leduc remarks that the heads of epileptics are larger and the skulls thicker than in healthy persons; an observation confirmed by Lorry and others. The predisposition to epilepsy, like that to many other diseases, is often hereditary, being transmitted from parent to progeny.

Almost all writers have placed plethora among the predisposing causes of this disease. We do not mean to say that general plethora is necessary; for we have seen epilepsy in every kind of temperament; but we are convinced from the phenomena during life, and the appearances *post mortem*, that *local* plethora exists in the head at the time, and probably before the attack commences. Of the exciting causes, some act directly on the brain, others produce their effects primarily upon distant parts, and secondarily upon the brain.

"Among those which act primarily upon the brain, the chief are mechanical causes, such as malconformation and injuries of the cranium, tumours, depressed bone, sharp pointed spiculæ, situated on the internal surface of the skull, hydatids, congestions distending the vessels of the brain, changes in its structure, or disease in its substance, and effusions of various kinds. To these may be added, causes acting mechanically on the spinal chord or the nerves, and the morbid appearances in the pineal gland and cerebellum, discovered after epilepsy by Mons. Wenzel, which have been already particularly described." 56.

The above causes, in all probability, act by stimulating or compressing some part or parts of the brain. M. Portal proved, by experiments, that pressure on the sensorium, in a great degree, will cause apoplexy; and, in a smaller degree, convulsions. Thus apoplexy, by a diminution of the power of the exciting cause, may end in epilepsy; and epilepsy, by an increase of it, may terminate in apoplexy—which indeed is not a rare occurrence. But we must pass over a great deal of valuable information collected by our author from various sources, because the subject of epilepsy occupied a good deal of our attention when reviewing Dr. Prichard's work in our last volume. We shall therefore pass on to the fifth and last chapter on the TREATMENT, occupying nearly two-thirds of the volume.

4. *Treatment.* Our author first notices the idiopathic species of epilepsy. Where premonitory symptoms are cognizable, as vertigo, pulsation of the carotids, confusion of intellect, and the like, then depletion should be immediately had recourse to—especially blood-letting and purging. Fraser, in his treatise on epilepsy, recommends 30 or 40 drops of laudanum in a drachm of camphor julep, to be taken as a preventive. The practice of stopping the progress of the aura epileptica to the brain by a ligature, was known to the ancients. We have little faith in such a process. In the actual paroxysm we can do little else than confine the patient, so as that he may not injure himself, loosening all his clothes, and keeping his head high, and preventing the laceration of the tongue by a piece of cork or wood in his mouth. The radical cure must be put in force during the intervals of the paroxysms. The correction of the predisposition, and the removal of the exciting causes, form, of course, the basis of the treatment; but the causes are often unknown, and in what the predisposition consists is often unknown also. As fulness of the cerebral vessels is one of the best ascertained conditions, as connected with epilepsy, the lowering plan is very generally recommended by both ancients and moderns —especially by Areteus among the former, and Tissot among the latter. Dr. Cooke introduces some observations and cases from Mr. Henry Earle which we shall here notice.

"A young gentleman," says Mr. Earle, "aged sixteen, was subject to epileptic fits, accompanied with most alarming determination of blood to the head. He dated these paroxysms from a fall which he had into the hold of a ship in China, where he subsequently suffered from a bad fever. The fits usually occurred before he rose in the morning; but he was frequently liable to them during the day. At the time when I first saw him, they were increasing so much in frequency, that he was never certain of passing a day without a seizure. He was constantly afflicted with severe head-aches, was very drowsy, and his memory was very defective. I was first called to him during one of the most severe attacks he had ever experienced. I found him perfectly insensible, with stertorous breathing, and his eyes and whole countenance evincing great determination to the head. He had passed his urine and fæces involuntary. I immediately bled him from the arm and jugular vein; but he did not evince any signs of returning sensibility until I had taken between forty and fifty ounces of blood. He remained in a state of stupor for some time, and experienced much numbness in his right arm and leg, which did not wholly subside for several days. I recommended a strict vegetable diet, and purged him freely, which kept off the attacks for some time. During the winter I was twice called to him, in nearly the same state as I have above described; each time the fit came on early in the morning, and required the same active measures to be pursued. From observing the very powerful action of the carotid arteries, I was induced, in one of his attacks, to try the effect of compressing those vessels, which appeared much to shorten the duration of the fit. I explained to him and his friends the exact situation of the vessels, and shewed him how to compress them, being anxious that he should make a trial of pressure, on the least threatening of a paroxysm, of the approach of which he was generally sensible from the rushing noise in his ears, and an indescribable dread which took possession of his mind. It was not long before an opportunity offered of making the experiment, and he was confident that he postponed the attack. Very frequently after this, he succeeded in arresting the approaching fits; but he was still occasionally liable to them early in the morning, on first waking." 114.

By perseverance in a very rigid diet and lowering remedies, he quite recovered. Mr. Earle has known another instance of epilepsy, in which pressure on the carotid arteries seldom failed to arrest the fit. He also witnessed a marked good effect from a local abstraction of blood in case of epilepsy of long standing.

"A lady, between thirty and forty, had been subject to fits from her infancy, for which she had been treated with strong stimulating nervous medicines, and very full diet. She generally experienced a succession of fits, to the extent of six or eight; and the attacks were always preceded and followed by severe head-aches, and much convulsive twitching. I found her just coming out of a second fit, and quite insensible; her countenance was crimson, and her eyes quite ferretty. The determination to the head was so marked, that I immediately recommended cupping from behind the ear, which was performed to the extent of sixteen ounces. She speedily became sensible, and had no return of fits; but instead of falling into a profound sleep, which was the usual consequence of an epileptic paroxysm, she became extremely talkative, and her imagination was so unusually active, that she soon began to speak as if conversing with persons who were not present. By reasoning with her, she was made sensible that she was talking incoherently; but soon relapsed again. I was called to visit her in this state, and by giving her a powerful dose of camphor and opium, she very soon became composed, and fell into a quiet sleep, from which she awoke perfectly well and free from head-ache. The effect of the sudden abstraction of blood from a surcharged brain in this case was very curious, and might lead to much speculation; but as facts are rather desired than theories, I give you the simple narrative." 117.

The foregoing case appears to have been complicated with some degree of that state which is denominated delirium tremens, or at least an approach to it. Out of five dissections which Mr. Earle has made of epileptic patients, two had tumours in the cerebellum and cerebrum—and three had "ossific productions from the basis oranii, which had induced chronic disease in the contiguous membrane and substance of the brain."

That blood-letting, local or general, is very generally a powerful auxiliary in the treatment of epilepsy, few will be inclined to deny; but we question whether more than a very few have been cured by this measure, or by it and the other means of depletion alone; where the cause is at a distance from the head, as for instance in children, where worms produce epilepsy, then purgation may cure the disease; but otherwise purgatives can only be considered in the light of auxiliaries. Setons or issues in the neighbourhood of the head or spine are more permanent depletors than purgatives, and may often be useful. Dr. Abercrombie is of opinion that the only remedies of real efficacy in these cases (plethoric epilepsy) are purgatives and strict vegetable diet, with total abstinence from strong liquors.

In opposite states of the system, tonics have been pretty generally exhibited by modern physicians, as bark, the misletoe of the oak, and metallic salts of various kinds. Dr. Fraser asserts that he cured nine cases out of eleven of epilepsy by the viscus quercinus. He gave it in powder in the dose of from two scruples to two drachms, twice a day, in a draught of camphorated emulsion. It is said, that the late Drs. Fothergill, Thompson, and Willan, gave this remedy also with success. It is now almost entirely neglected.

The nitrate of silver is the empirical remedy at present most in use for the cure or mitigation of epilepsy, and Dr. Cooke has collected a considerable mass of living authorities on the subject. Dr. Baillie has a high opinion of this medicine, and frequently prescribes it. Dr. Powel has long ago proved that it may be taken in far greater doses than one would be led to suppose from its effects on the external surface of the body.

It is now pretty generally known that the nitrate of silver, when taken a certain length of time, will, in some constitutions, turn the skin of a blue colour. We believe the late Mr. Goldson of Portsmouth was the first to notice this curious fact, about twenty or more years ago. The lady who was cured of epilepsy by him is, we believe, still living, with a darkened complexion. There are now some thirty or forty instances on record of a similar kind—and in the majority of these cases, the disease was subdued, which is some compensation for the change of complexion. We have only seen two instances of the kind, and in both the cure was perfect. From all the enquiries we have been able to make, no case has occurred where this discoloration took place under *four months*' use of the nitrate. We believe Dr. Baillie makes it a rule not to continue the medicine longer than the above mentioned period at any one time. Dr. Roget has informed Dr. Cooke that he has known *several instances* of this discoloration without any cure of the disease. In the instance, however, which he published in the 7th vol. of the Medico-Chirurgical Transactions, the cure remains complete, as well as the blue colour, after an interval of twelve years.

The preparations of Zinc are by some considered efficacious in epilepsy, and so has been the cuprum ammoniatum, especially by Cullen. Our readers are aware that Dr. Shearman has recommended the "elutriated oxyd of tin," as a remedy of much service in this disease. Dr. Abercrombie informs our author that he has seen very good effects from keeping patients under the influence of tartrite of antimony, in such doses as the stomach could bear, repeated four times a day. The modus operandi of these medicines is but little known. The oil of turpentine has lately been a good deal used in epilepsy. Dr. Latham, in his work on diabetes, informs us that he has several times relieved, and more than once cured epilepsies by the oleum terebinthinæ. A great many testimonies to the anti-epileptic properties of this medicine are now to be found scattered through our periodical publications. Dr. Young, in the 5th volume of the College Transactions, gives two instances of the good effects of the oleum terebinthinæ rectificatum, exhibited in large doses. The late Dr. Edward Percival, of Dublin, as our readers know, has published some interesting observations on this remedy in maniacal epilepsy, as the result of much experience in the Hardwicke Lunatic Asylum. The form used was this :—an ounce of the oil was triturated with an equal weight of lump sugar, and a pint of spearmint water was gradually added. A full dose of this emulsion is an ounce three times a day. The salutary effects of this medicine in the cases detailed were very evident; for besides the striking alleviation of their epileptic disorder, and its maniacal adjuncts, all of the patients gained considerable improvement in their general health, appetite, and cheerfulness. Mr. Lithgow of Coleraine has also given us a strong testimony in favour of the medicine. Our readers are aware that Dr. Prichard is a strenuous advocate for this remedy in uterine and enteric epilepsy, as was seen in our ninth number.

Of the administration of opium, hyoscyamus, digitalis, and other sedatives, we need not speak, as few men, now-a-days, put any confidence in them. In a former number of this journal we gave some account of Mr. Mansford's work on epilepsy, and of his apparatus for carrying off the excess of electric matter from the brain, in which consists, he thinks, the proximate cause of epilepsy. We do not suppose that this excentric theory of Mr. Mansford has made any impression on the profession, and we should not have noticed it here, had it not been that we lately came across an old acquaintance who had long been subject to epilepsy, and who adopted the apparatus as described in Mr. Mansford's work, with success. Whether the force of imagination had any thing to do in this case, or that the cure was owing simply to the remedy, we do not profess to decide—we only relate the fact as it has come to our knowledge.

"In some instances," says Dr. Cooke, "both the cautery and trephine appear to have been employed by the antients in attempting the cure of epilepsy; but the moderns, until very lately, did not venture to try the former of these powerful remedies. The operation of trepanning was, however, recommended in some cases, by Boerhaave and others of his time." 195.

Of late years the ancient practice of cauterization has been revived—particularly by Baron Percy and Dr. M. Gondret. In the Baron's work (Pyrotechnie Chirurgicale) we have a very minute account of every thing relating to the use of the actual cautery in medicine and surgery. He agrees with Galen and Areteus that a very hot cautery is, to one of a lower degree of heat, so far as relates to pain, what a very sharp bistoury is to one that is blunt. He says that the cauterization of bones gives no pain.

"The success of the application of fire, which Baron Percy has witnessed in many terrible cases of epilepsy, he refers to the simultaneous cauterization of the skin, the pericranium, and the bone, exciting in the cerebral system that specific commotion, that sensation from fire (sensation ignée) which no other agent can produce." 199.

The ulceration and discharge, he says, must be kept up, after the cautery, for a considerable time, if we hope to reap the full benefit of the operation.

Dr. Gondret has also employed, with great success he avers, the actual cautery in many cases of epilepsy supposed to depend on a diseased state of the cranium, brain, or membranes. He adduces several striking instances of the great utility of this practice, and promises to bring forward many others. But although M. Gondret had completely convinced himself of the truth of his doctrines and efficacy of his practice, he found great difficulty in persuading his patients to submit to operations so abhorrent to the feelings. He therefore directed his attention to the discovery, (if possible) of a substitute for the actual cautery.

"What M. Gondret wanted was an application which might either irritate, or produce a rubefacient, or a vesicating, or an escharotic effect, according to the indications to be fulfilled. This, he says, he was fortunate enough to discover in a simple preparation, well known and much used, though for a different purpose, namely ammonia. This alkali, joined with fat or olive oil, he found would form a pomade capable of fully answering his purpose, by acting as a stimulus in different degrees; so that when slightly rubbed on the surface, it produced gentle irritation of the skin; when spread on linen and applied for six or eight minutes it acted as a rubefacient; when for a quarter or half an hour, it occasioned vesication; and if applied for a still longer time, it proved escharotic." 203.

The form which M. Gondret uses is six drachms of suet, one or two tea spoonfuls of oil of almonds, and one ounce of the liquor ammoniæ puræ.

Upon the whole, as Dr. Cooke properly observes, although this treatment (the actual and potential cautery) may give much pain and inconvenience, yet it promises considerable benefit, in epilepsies dependent on or connected with malconfirmation of the cranium, exostoses, depressed bone, a diseased state of the investing membranes, or any cause acting mechanically upon the surface of the brain.

On the epilepsies connected with, or sympathetic of, certain morbid affections of the abdominal or pelvic viscera, particularly of the intestines and uterus, Dr. Cooke offers very judicious observations and directions; but as these have been fully treated of in our review of Dr. Prichard's work, we must here pass them over, referring to Dr. Cooke's excellent epitome of ancient and modern opinions upon all points connected with the disease. We need not here repeat the opinion which, on several occasions, we have been induced to offer on the merits of these volumes. But we can bear testimony to the truth and accuracy of the concluding passage in Dr. Cooke's publication.

"I have now finished what I had to communicate respecting the history, causes, and method of cure, of apoplexy, palsy, and epilepsy.—In my account of these very important nervous diseases, I have endeavoured to abstract, to condense, to methodize, and to convey in clear and plain language, the best information I could collect from a great number of writers, both antient and modern.—I cannot flatter myself that, by the investigation which I have made of these obscure disorders, I have done much towards the illustration of their nature; but I do hope that the description I have given of the experiments, observations, opinions, and practice, of the most celebrated physicians in various ages, respecting them, will prove, in some degree, useful, both by lessening the labours of the student, and by affording practical assistance to persons who are actually engaged in the duties of the profession." 235.

The plan and professed object of Dr. Cooke's work necessarily precluded the attempt to introduce any novelty or much original matter on his own part. A comment or occasional remark on the opinions of others, with short statements of his own experience, were all that he could introduce into a work of this kind; and these he has introduced—sparingly it is true, but always judiciously. We take leave of our author for the present, with every sentiment of respect and esteem.

